# Special Issue: “Emerging Therapies and Strategies in Thalassemia: Toward a New Era in Management”

**DOI:** 10.3390/jcm11175175

**Published:** 2022-09-01

**Authors:** Paolo Ricchi, Gian Luca Forni

**Affiliations:** 1 Center for Rare Red Blood Cell Diseases, AORN A. Cardarelli, 80131 Naples, Italy; 2 Center for Congenital Anemias and Iron Dysmetabolism, Galliera Hospital, 16128 Genoa, Italy

This Special Issue on “Emerging Therapies and Strategies in Thalassemia: Toward a New Era in management” aims to update researchers and clinicians regarding the field of thalassemia syndromes. Out of the seven articles published in this Special Issue, two are manuscripts that provide information on the strategy of chelation therapy in transfusion-dependent thalassemia (TDT) patients from the real-life context of two Italian centers for hemoglobinopathies comprehensive care. These studies evaluated two opposite conditions. The first article by Ricchi et al. reported a cross-sectional study and focuses on the feasibility of achieving serum ferritin < 500 ng/mL for several TDT patients under different chelation regimens [1]. Such a ferritin target, at MRI T2* evaluation, was associated with the absence of significant cardiac and hepatic iron overload (IO) but with a noteworthy proportion of patients showing simultaneous pancreatic IO. Therefore, the pancreas appeared as a “sanctuary compartment” for common iron-chelating agents. Prospective assessments are required to establish the benefit of removing pancreatic IO in TDT.

By contrast, in the article by Origa et al., the effects of new combined therapies were retrospectively evaluated in TDT patients with high serum ferritin values and severe or moderate liver IO, refractory, intolerant or not adherent to previous chelation regimens [2]. In particular, they assessed the safety and efficacy of long-term combinations of iron-chelation regimens with deferasirox (DFX) plus desferrioxamine (DFO) and DFX plus deferiprone (DFP). Both treatment regimens reduced serum ferritin and liver iron concentration measured by MRI as R2*. However, only in those treated with DFX plus DFP did the cardiac iron overload significantly decrease. The safety of both combinations was consistent with established monotherapies. A useful treatment algorithm for patients with TDT with severe iron burden, or for those where monotherapy is not adequately effective, it is also proposed. Authors concluded that larger, multicenter studies are needed to shed further light on the advantages and limitations of the simultaneous use of DFO and DFX and two oral chelators.

Generally, a better explanation of the pathway of organ iron overload and clearance in the different organs by MRI could significantly contribute to the better management of all thalassemic patients. That pancreatic iron overload is an intense and stimulating area of investigation was made evident in the article by Meloni et al. [3]. They explored, for the first time, the prevalence of pancreatic IO in thalassemia intermedia (regularly and non-transfused patients) and the link between pancreas T2* values and glucose metabolism and cardiac outcomes. They found both that pancreatic IO increases with regular transfusions and that there was an association between HCV infection, splenectomy and higher levels of pancreatic iron deposition. They also confirmed the previous observation in TDT patients that a normal global pancreas T2* value showed a negative predictive value of 100% for cardiac iron and for dysregulated glucose metabolism; therefore, this study reinforces the importance of quantifying iron status at pancreatic level in thalassemic patients. However, further studies are needed to clarify the requirements for initiating or intensifying iron chelation therapy based on pancreatic IO to prevent the alteration of glucose metabolism.

Other features of the management of thalassemic patients are also addressed in this Special Issue. In the review by Longo F. and Piga A., an accurate and updated step by step evaluation of pathophysiological mechanisms underlying iron overload and ineffective erythropoiesis in beta thalassemia patients is presented [4]. The review describes in detail new strategies and those that are currently in development, which can inhibit these unsettled mechanisms in thalassemia syndromes. In particular, they focus on targeting the hepcidin-ferroportin pathway as a novel approach to achieve further control over disease burden.

The last review published in this Special Issue is an original and updated review by Tartaglione et al. [5]. The authors present the first systematic review of all the published studies via a hearing assessment in β thalassemia. They evaluated hearing impairment in patients with thalassemia in order to define its prevalence, features, course, and possible disease- or treatment-related pathogenic factors. Following an evaluation of all the collected articles, they concluded that future longitudinal studies with a detailed description of sample, treatment, and hearing deficit will help understand the pathogenesis, prevalence, and best management strategy for hearing impairment caused by beta thalassemia.

The last article of this Special Issue is a formal document, entitled “Good Clinical Practice of the Italian Society of Thalassemia and Haemoglobinopathies (SITE) for the Management of Endocrine Complications in Patients with Haemoglobinopathies” [6]. Endocrine complications are the most frequent and most resource-draining complications in haemoglobinopathies. Therefore, The Italian Society of Thalassemia and Haemoglobinopathies (SITE) has undertaken a project that aims to integrate the available evidence with experts’ opinions through a systematic method, in order to obtain an adequate degree of consensus on the recommendations in clinical practice. This type of indication is fundamental, especially if there is insufficient evidence for an evidence-based recommendation, and the panel considers it important to provide such a recommendation. The main part of the document focuses on transfusion-dependent thalassemia, due to the higher frequency of endocrine disorders in this group of patients. Recommendations related to endocrine disorders in non-transfusion-dependent thalassemia and sickle cell disease are reported in a specific chapter at the end of the document.

In conclusion, we are aware that this Special Issue has covered only limited features regarding the therapeutic advance in the management of patients with thalassemia; however, we hope that readers will find our published articles informative in order to encourage future research ideas and carry out more studies in the broad field of thalassemia syndromes, where much remains to be discovered.
